# Augmented Reality in Surgical Training: Systematic Review of Its Impact on Technical Performance in Surgical Trainees

**DOI:** 10.2196/71572

**Published:** 2026-06-03

**Authors:** Mahmoud El Ashry, Ahmed El Ashry, Hamza Khalique, Yahye Abdalle, Thomas Yeung

**Affiliations:** 1 Bristol Medical School, University of Bristol, Beacon House, Queens Road, Bristol, BS8 1QU, United Kingdom, 44 7448827361; 2 ENT/Otolaryngology Department, University Hospitals of North Midlands NHS Trust, Stoke-on-Trent, United Kingdom

**Keywords:** augmented reality, surgical education, systematic review, head-mounted display, simulation, PRISMA, telestration, skill acquisition

## Abstract

**Background:**

Surgical training has changed over the past decade. Augmented reality (AR) has become one of the more talked-about developments within that space. At its core, AR works by placing digital information over the real-world environment. This gives trainees guidance and spatial cues during a procedure as they perform it. What remains uncertain is whether AR moves the needle on technical skill development in trainees. The studies that address this directly are few, and the ones that do exist rarely speak to each other in any meaningful way. Outcome measures shift from paper to paper, the hardware studied spans a wide range of maturity, and methodological consistency is hard to find.

**Objective:**

This systematic review assesses the impact of AR on the objective technical skills of surgical trainees when compared with traditional methods.

**Methods:**

We searched PubMed, MEDLINE, Embase, IEEE Xplore, Scopus, and Web of Science for studies published between January 1, 2020, and September 15, 2025. From 4799 initial records, 1417 remained after deduplication. Of these, 101 underwent detailed abstract review and 29 were assessed in full text. Eleven studies met the inclusion criteria. Two reviewers (MEA and YA) independently screened all records, with a third senior reviewer (TY) resolving disagreements. We performed a narrative synthesis following SWiM (Synthesis Without Meta-Analysis) guidelines across 5 thematic domains to account for study heterogeneity.

**Results:**

The final analysis included 11 studies (347 participants across 7 specialties) published between 2021 and 2025. These included 9 randomized controlled trials and 2 prospective cohort studies. The studies used platforms such as the Microsoft HoloLens (1 and 2), Magic Leap One, and Vuzix M300XL. Of the 11 studies, 9 reported improvements in one or more objective technical metrics. Key findings included consistent error reduction (5/5 studies), faster learning curves (4/11 studies), and lower cognitive workload (3/11 studies). Notably, an “expertise reversal” effect was observed, where AR provided substantial benefits to novices but diminishing returns for experienced surgeons.

**Conclusions:**

AR significantly improves technical performance for surgical novices, particularly in tasks involving complex visuospatial reasoning. AR is an effective tool in surgical education. Future research should focus on multicenter trials to evaluate long-term skill retention and cost-effectiveness in clinical practice.

## Introduction

Surgical training has transformed over the past two decades. The erosion of the traditional apprenticeship model, driven by reduced working hours, patient safety concerns, and the expansion of minimally invasive techniques, has created an urgent need for high-fidelity, reproducible alternatives to case-based learning [1,2]. The consequences of this shift are well documented: surgical residents are entering independent practice with fewer operative experiences than their predecessors, and the early learning curve, particularly in complex and high-stakes procedures, carries measurable risk to patients [3,4]. Thus, simulation-based surgical education has emerged as a necessary complement to the operating room, and the last decade has witnessed extraordinary investment in the development and evaluation of digital training modalities [5,6].

**Figure 1.**
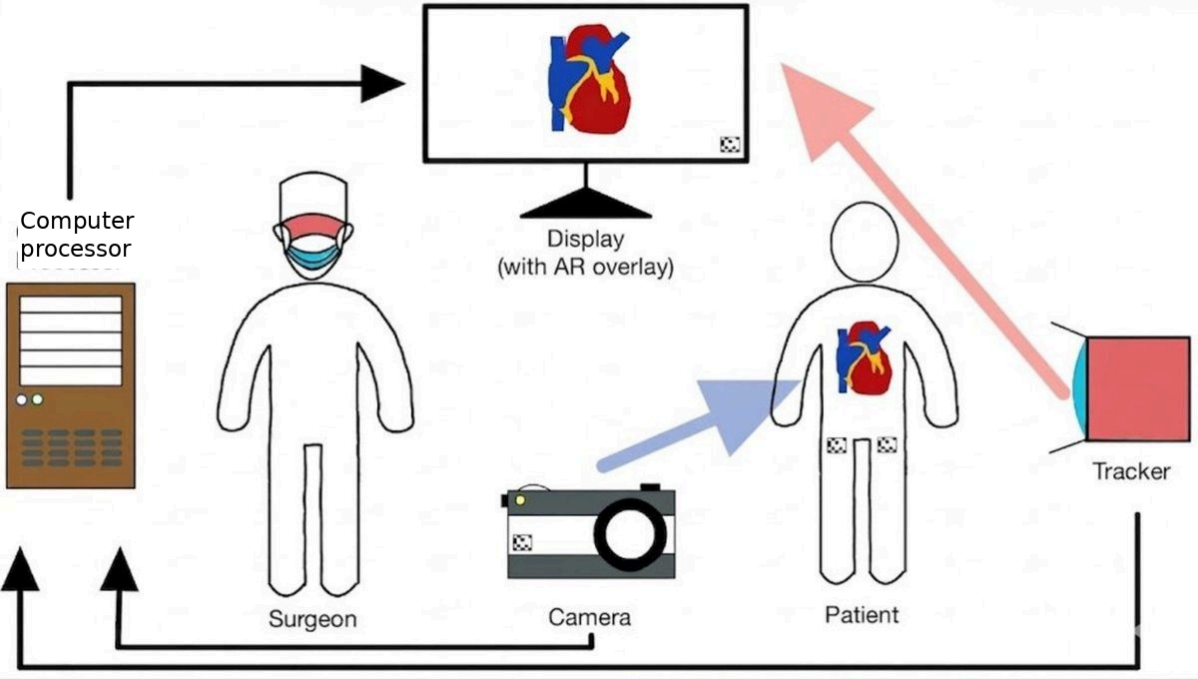
Schematic diagram illustrating the basic principles of augmented reality as applied in a surgical context: a camera captures the physical operative environment; a computer processor generates a digital overlay (eg, anatomical structures, procedural guidance, or expert telestration); the overlay is projected onto a display visible to the surgeon, while a tracker provides positional feedback for accurate registration of virtual content to the physical world. AR: augmented reality.

Virtual reality (VR) and augmented reality (AR) have particularly attracted attention among users. Both AR and VR rely on immersive technology to create a virtual surgical learning environment for students. However, they differ fundamentally in their relationship to the physical world. VR relies solely on a synthetic environment, keeping the student separate from real-world stimuli, and has demonstrated efficacy in improving laparoscopic and robotic surgical skills across several procedural domains [7,8]. AR, by contrast, overlays computer-generated information, such as anatomical structures, navigational guides, procedural annotations, or expert telestration, directly onto the user’s real-world field of view through optical head-mounted displays (HMDs), smart glasses, or screen-based systems [9,10]. This technical workflow, as illustrated in Figure 1, relies on a closed-loop system where physical data is captured, processed, and reprojected as a digital overlay in real time. These AR modalities maintain contact with physical instruments, simulators, and task environments while simultaneously providing contextually embedded guidance. The relative merits of VR and AR in surgical training remain an active area of investigation; the two modalities address different cognitive and technical challenges and are most productively viewed as complementary rather than competitive [11]. Mixed reality (MR), exemplified by platforms such as the Microsoft HoloLens, extends AR by enabling dynamic interaction between virtual and physical objects through spatial mapping, though the boundary between AR and MR in the surgical training literature remains inconsistently defined. While the boundary between AR and MR is often inconsistently defined in broader literature, this review strictly defines AR as the unidirectional overlay of digital data onto a physical field. We explicitly exclude bidirectional, spatially mapped “mixed reality” interactions to isolate the cognitive impact of the digital overlay itself.

The theoretical basis for AR’s educational value is well established. Cognitive load theory posits that learners have finite working memory capacity and that effective instruction should minimize extraneous cognitive load while maximizing germane load, the mental effort directed toward schema formation [12]. Conventional verbal instruction in minimally invasive surgery is particularly demanding, requiring trainees to mentally translate auditory descriptions into spatial awareness of the operative field in real time. AR telestration and anatomical overlay systems bypass this cognitive translation step by providing visual guidance precisely colocated with the task being performed, theoretically reducing extraneous load and accelerating the formation of procedural schemas [13]. The expertise reversal effect predicts that while AR provides essential scaffolding for novices (maximizing germane load), these same overlays may function as extraneous cognitive load for experts who have already developed robust internal schemas, potentially hindering rather than helping performance [14].

Since 2020, published evidence for AR usage has expanded significantly, but despite this, existing systematic reviews have significant limitations: they frequently analyze AR, VR, and MR simultaneously or separate them with loose boundaries, and sometimes include older prototype systems. Studies also rely predominantly on subjective or process-based outcomes rather than objective technical performance metrics [15,16]. No contemporary synthesis has focused exclusively on AR’s impact on trainee technical performance using validated, objective outcome measures across the most recent generation of AR technology. This study aims to achieve this while also maintaining strict boundaries surrounding the inclusion of solely AR-based projects for review.

This systematic review addresses that gap. Using a prespecified PICO (population, intervention, comparison, outcome) framework: surgical trainees (population), AR-based training interventions (intervention), traditional surgical teaching methods (comparison), and objective measures of technical performance (outcome), we synthesize contemporary evidence from 2020 to 2025 to answer the question: does augmented reality, when used as an adjunct to or replacement for traditional surgical training, improve objective technical performance in surgical novices? By confining our scope to studies with defined AR interventions, controlled comparators, and objective outcome measures, this review provides a focused, methodologically rigorous assessment of AR’s current standing as a surgical training modality.

## Methods

### Study Design and Registration

This systematic review included a literature search and a write-up that were both carried out with respect to the PRISMA (Preferred Reporting Items for Systematic Reviews and Meta-Analyses) 2020 statement [17]. The completed PRISMA 2020 expanded checklist is provided as Checklist 1. A narrative synthesis was performed in accordance with the Synthesis Without Meta-Analysis (SWiM) reporting guideline [18]. This type of analysis was chosen as significant clinical and statistical heterogeneity existed across included studies making statistical pooling and group analysis of studies unrealistic. The approach is consistent with the methodological guidance of the *Cochrane Handbook for Systematic Reviews of Interventions* [19]. Searches were additionally reported in accordance with the PRISMA-S extension for the reporting of literature searches in systematic reviews [20]. The review was not prospectively registered. However, an a priori protocol specifying the research question, eligibility criteria, databases, search strategy, data extraction variables, and planned synthesis approach was developed and followed throughout.

### Eligibility Criteria

Eligible studies for analysis had to be original, peer-reviewed articles published in English, with a publication window between January 1, 2020, and September 15, 2025. Participants could be trainees at any stage of their training. This meant if participants of studies were medical students, residents, and fellows, the study was appropriate for inclusion.

On the intervention side, a clearly described augmented reality component was required. For the purposes of this review, AR was understood as technology that overlays digital content directly onto a person’s view of the real world. Since AR, VR, and MR tend to appear together frequently in the literature, screening by YA and MEA was agreed beforehand to be meticulous and deliberate in only including studies that clearly focused on independently analyzing AR only. Each study also needed to include a comparator condition, whether that entailed traditional instruction, conventional operative guidance, or a freehand approach. At least one objective measure of technical performance or skill acquisition had to be reported (Table 1).

**Table 1.** Inclusion and exclusion criteria for study eligibility.

Category	Inclusion criteria	Exclusion criteria
Publication	Peer-reviewed original research; published after January 1, 2020; English language	Reviews, editorials, letters, conference abstracts; published before 2020; non-English
Participants	Human surgical or procedural trainees (medical students, residents, and fellows) at any training stage	Expert surgeons performing clinical procedures without a trainee component; nonmedical participants
Intervention	Clearly described AR^a^ component overlaying digital information onto real-world view; outcomes attributable to AR isolable from VR^b^/MR^c^	AR inseparable from VR or MR; purely passive AR use without guidance or training function; technology validation without trainee performance outcomes
Comparator	Traditional instruction, verbal guidance, conventional teaching, or freehand technique	No comparator or control condition present
Outcomes	At least one objective measure of technical performance (eg, accuracy, error count, validated skill score, and procedure time)	Outcomes entirely subjective (questionnaire only); usability/feasibility data only; no measurable performance data reported

a AR: augmented reality.

b VR: virtual reality.

c MR: mixed reality.

Several categories of studies were excluded from the outset. Work published before 2020 was not considered, nor were non-original outputs such as reviews, editorials, or conference abstracts. Studies conducted exclusively with expert surgeons performing live clinical procedures fell outside the scope unless a formal training element was present. Where AR could not be disentangled from VR or MR, or where all reported outcomes were subjective in nature, studies were similarly excluded—as were those lacking any comparator.

The 2020 cutoff was a deliberate methodological choice rather than an arbitrary date. AR hardware and software underwent substantial development around 2018‐2019, and a good deal of earlier work was conducted using prototype or near-prototype systems that bear little resemblance to the tools in use today. Including that literature risked drawing conclusions that would not generalize meaningfully to contemporary training contexts, so it was excluded on those grounds. The 2020 cutoff marks the transition from prototype-based research to the use of enterprise-grade, high-fidelity hardware (eg, Microsoft HoloLens 2 and Magic Leap One). Including earlier data from low-resolution prototype systems would introduce technological bias and yield conclusions that do not generalize to contemporary surgical training environments.

### Information Sources

Six electronic databases were searched: PubMed (MEDLINE), Ovid MEDLINE, Embase, IEEE Xplore, Scopus, and Web of Science. The selection was deliberate rather than exhaustive for its own sake. AR in surgical training sits at the intersection of clinical medicine and engineering, and no single database captures that breadth adequately—so the combination was chosen to reflect it. PubMed, Ovid MEDLINE, and Embase covered the biomedical literature, with Embase included specifically because its indexing patterns differ enough from PubMed to reduce the risk of missing relevant work, consistent with *Cochrane Handbook* recommendations on database selection [19]. IEEE Xplore addressed the engineering and technology side of the literature, where much of the platform development and human-computer interaction research is published. Scopus and Web of Science brought broader multidisciplinary coverage across both domains.

Google Scholar was not included in the primary database search. Reasons for this included the search base’s nontransparent indexing algorithm, inclusion of non–peer-reviewed sources, and absence of a reproducible search interface. These issues would result in the search falling short of the reproducibility standards required by PRISMA and the *Cochrane Handbook,* which we aimed for [19,21]. The search across all 6 databases was executed on April 24, 2026, with results filtered to the eligibility window of January 1, 2020, through September 15, 2025. Reference lists of relevant systematic reviews identified during screening were then hand-searched to capture any eligible work that database searching alone might have missed.

### Search Strategy

Our search strings were drafted by combining Medical Subject Headings (MeSH) terms with free-text keywords, referring to the PICO framework. We also reflected on guidance from Chapter 4 of the *Cochrane Handbook* [19]. There were 3 domains in particular that formed the backbone of the search strategy: the technology itself (augmented reality), the population and setting (surgical or procedural trainees), and the outcome domain (technical performance or skill acquisition). Boolean operators (AND, OR) were then used to combine terms within search strings. Truncation with wildcards was applied where database syntax allowed. The full PubMed search string is provided in Textbox 1.

Textbox 1. **Full PubMed search string.**

(“Augmented Reality”[MeSH] OR “augmented reality”[tiab] OR “mixed reality”[tiab] OR “head-mounted display”[tiab] OR “HMD”[tiab] OR “heads-up display”[tiab] OR “HUD”[tiab] OR “holographic”[tiab] OR “telestration”[tiab] OR “HoloLens”[tiab] OR “Magic Leap”[tiab] OR “smart glasses”[tiab] OR “optical see-through”[tiab] OR “AR-assisted”[tiab] OR “AR-guided”[tiab] OR “AR-enhanced”[tiab]) AND (“Education, Medical”[MeSH] OR “Education, Medical, Graduate”[MeSH] OR “Clinical Competence”[MeSH] OR “Simulation Training”[MeSH] OR “Internship and Residency”[MeSH] OR “surgical train*“[tiab] OR “surgical educat*“[tiab] OR “surgical skill*“[tiab] OR “surgical simulat*“[tiab] OR “procedural train*“[tiab] OR “procedural skill*“[tiab] OR “resident*“[tiab] OR “novice*“[tiab] OR “trainee*“[tiab] OR “medical student*“[tiab] OR “laparoscopic train*“[tiab] OR “minimally invasive train*“[tiab] OR “neurosurgery train*“[tiab]) AND (“learning curve”[tiab] OR “technical performance”[tiab] OR “skill acquisition”[tiab] OR “psychomotor”[tiab] OR “accuracy”[tiab] OR “proficiency”[tiab] OR “competency”[tiab] OR “OSATS”[tiab] OR “GOALS”[tiab] OR “error rate”[tiab] OR “performance score”[tiab] OR “procedure time”[tiab]) AND (“2020/01/01“[PDat]:”2025/09/15”[PDat])

This search strategy was adapted for the syntax and controlled vocabulary of each database. For IEEE Xplore, MeSH terms were replaced with IEEE Thesaurus terms if appropriate. For Scopus and Web of Science, equivalent field tags (TITLE-ABS-KEY) were used with the same conceptual terms. For Ovid MEDLINE and Embase, the Ovid MeSH explode function was used to capture all relevant subheadings. Full search strategies for all 6 databases are provided in Multimedia Appendix 1, reported in line with the PRISMA-S guideline [20].

A data restriction of January 1, 2020, to September 15, 2025, was applied across all databases. No language filter was applied at the search stage itself. We felt that by restricting the language at that point, we risked inadvertently suppressing relevant records before they could be assessed. Non-English records that did not come through were excluded at screening. Similarly, no publication type or study design filters were applied during the initial search. This was on the basis that this runs the risk of missing eligible studies that could be indexed or tagged inconsistently across databases.

### Study Selection

All records retrieved from the 6 databases were imported into Rayyan (Qatar Computing Research Institute) for deduplication and screening. Rayyan automatically identified potential duplicates, which were manually verified and removed, leaving 1417 unique records for screening. Study selection was then conducted in 3 sequential stages by two independent reviewers (MEA and YA). In the first stage, titles of all 1417 unique records were screened against the prespecified eligibility criteria, reducing the pool to 101 records. In the second stage, the 101 records were reviewed through a detailed assessment of their abstracts against the full eligibility criteria to determine if they could pass onto the next stage. In the third stage, full texts of the 29 records were retrieved and assessed independently for final inclusion. Records for which full text could not be obtained via institutional access or interlibrary loan were pursued via direct author contact before being counted as inaccessible. Discrepancies at all stages were resolved through discussion and consensus between the two reviewers. If consensus could not be reached, a third senior reviewer (TY) acted as adjudicator. The reasons for exclusion at each stage are documented and reported in the PRISMA flow diagram.

### Data Collection Process and Items

Data extraction was carried out independently by two reviewers (MEA and YA) using a prestandardized form built in Microsoft Excel. Before the main extraction began, the form was piloted on two studies and adjusted where wording was ambiguous, or fields needed refinement. This was a small but useful step that avoided inconsistencies surfacing later in the process.

The form captured 7 categories of information: study identification details (first author, year, country, and journal); study design; participant characteristics, including specialty, training level, total sample size, and group allocation; details of the AR intervention, covering the specific platform and device used, what the AR content actually consisted of, and how it was delivered; comparator characteristics; the task or procedural scenario; and all reported outcomes measures including primary and secondary endpoints alongside their associated statistical results, including means (SDs), *P* values, and effect sizes if reported.

Responsibility for extraction was shared equally between MEA and YA. If the two reviewers disagreed on whether a data point should be included, they discussed this together with adjudication by a third reviewer (TY) if no consensus could be reached.

### Risk of Bias Assessment

Risk of bias was assessed independently by two reviewers (MEA and YA) using a tool selection determined by the study design. Randomized controlled trials (RCTs) were assessed using the Cochrane Risk of Bias 2 (RoB 2) tool [22]. Nonrandomized prospective cohort studies were assessed using the Risk of Bias in Nonrandomized Studies of Interventions (ROBINS-I) tool [23]. Crossover randomized trials were assessed using the RoB 2 tool with the crossover extension, as crossover designs remain RCTs and ROBINS-I is not applicable to them. Disagreements between reviewers were resolved by discussion; unresolved disagreements were adjudicated by the third reviewer (TY). Risk-of-bias findings are presented narratively in the Results section and considered in the interpretation of the overall body of evidence.

### Synthesis Methods

Given the substantial clinical heterogeneity across included studies in terms of AR platforms used, participant training levels, surgical specialties, comparator conditions, and outcome measures used, statistical pooling (meta-analysis) was not appropriate and was not performed. No standardized effect sizes (eg, Cohen *d* and standardized mean difference) were reported by included studies in a sufficiently consistent form to enable pooling; where individual studies reported effect sizes, these are noted in the narrative synthesis. Findings were synthesized narratively following the SWiM reporting guideline [18]. Studies were grouped according to five prespecified thematic outcome domains derived during the protocol stage: (1) technical accuracy and procedural performance, (2) error reduction and procedural safety, (3) learning trajectory and skill acquisition, (4) cognitive load and gaze efficiency, and (5) operational efficiency and procedure time. Within each domain, consistency and heterogeneity of findings across studies were assessed narratively, and the direction and magnitude of effects were described in relation to methodological quality.

## Results

### Study Selection

The systematic search returned 4596 records across the 6 databases (Scopus: 1640; Web of Science: 1039; IEEE Xplore: 790; Embase: 638; Ovid MEDLINE: 438; PubMed: 51). A further 203 records came from reference list checking and gray literature searching, bringing the total to 4799. Rayyan automatically identified 3382 potential duplicates, which were manually verified and removed, leaving 1417 unique records for screening.

MEA and YA independently screened all 1417 records by title, excluding 1316. The most common reasons were: no AR component or AR not separable from VR/MR (n≈478), no objective performance outcome (n≈369), not involving surgical or procedural trainees (n≈210), not an interventional study design (n≈171), no comparator group (n≈85), and non-English language (n≈3). Disagreements were resolved by consensus. The remaining 101 records proceeded to a detailed abstract review.

Both reviewers independently assessed all 101 abstracts, excluding 72. The main reasons were technology development or validation studies with no trainee performance outcomes (n=26), no clearly defined novice or trainee population (n=18), outside scope (n=14), non-original research formats including systematic reviews and editorials (n=8), and nontrainee participants (n=6). Where the two reviewers could not reach agreement through discussion, TY adjudicated. Twenty-nine records were then retrieved for full-text assessment.

**Figure 2.**
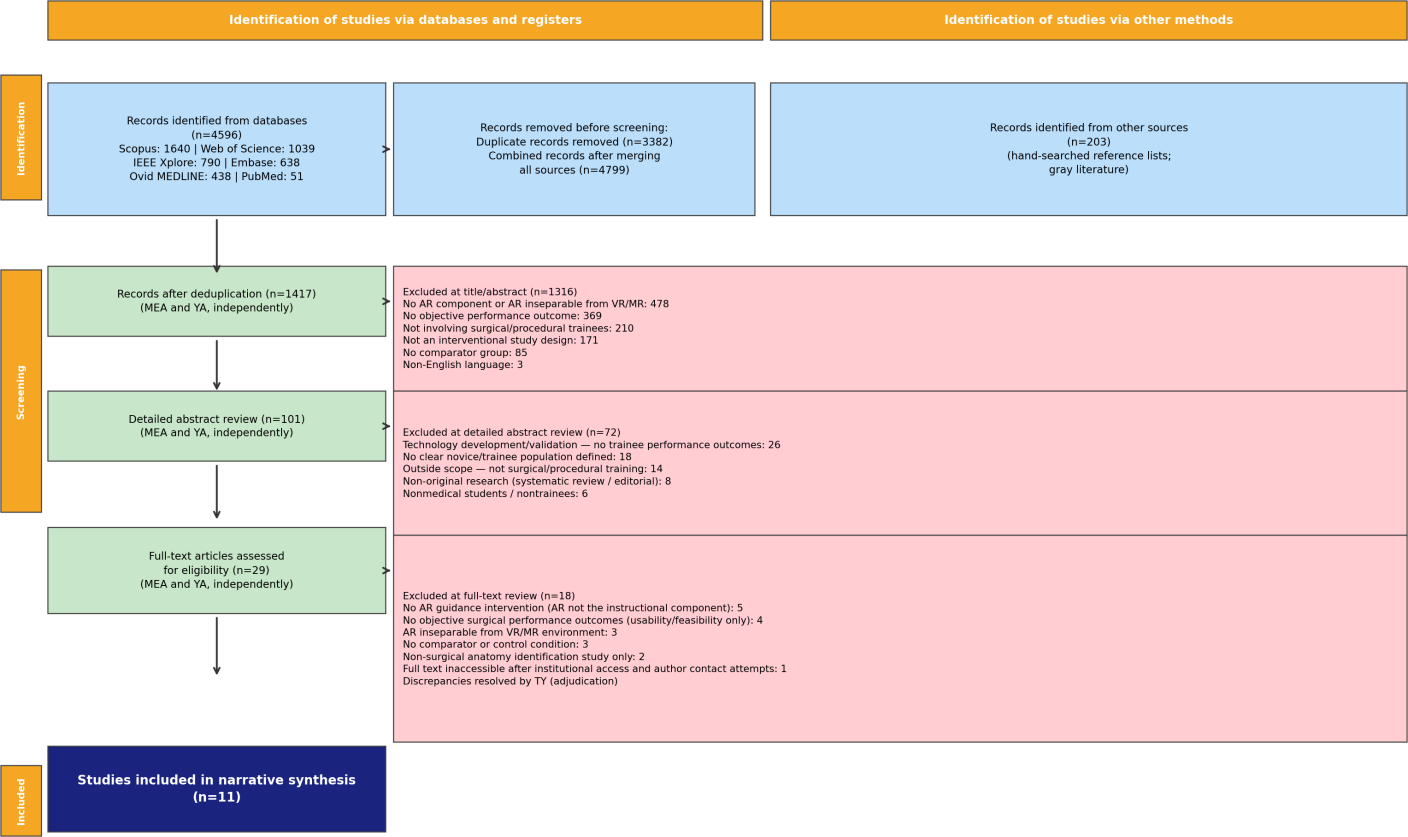
PRISMA 2020 flow diagram illustrating the study selection process. Records were identified across 6 databases and additional sources. Sequential screening at title/abstract, detailed abstract review, and full-text stages progressively narrowed the pool from 4799 records to 11 included studies. AR: augmented reality; MR: mixed reality; VR: virtual reality.

At full-text review, again conducted independently by MEA and YA, 18 articles were excluded. Reasons were: AR present in the setting but not functioning as the instructional component (n=5); usability or feasibility assessment only, with no objective performance outcomes (n=4); AR inseparable from a VR or MR environment (n=3); no comparator or control condition (n=3); anatomy identification study without any surgical skill training component (n=2); and full text inaccessible despite institutional access attempts, an interlibrary loan request, and direct contact with the authors (n=1). Disagreements were again resolved by discussion, with TY available for adjudication where needed. Eleven studies met all prespecified eligibility criteria and were included in the final narrative synthesis. The full selection process is shown in the PRISMA 2020 flow diagram (Figure 2).

### Study Characteristics

The 11 included studies span 2021 to 2025 and together enrolled 347 participants. Nine were RCTs—5 parallel-group [3,5,6,8,9], 3 crossover [1,4,7], and 1 three-arm [11], with the remaining 2 being prospective cohort comparisons [2,10]. Sample sizes ranged from 8 (Liu et al [2]) to 60 (Wild et al [7]). Across all studies, participants had no or very limited prior experience in the procedure being trained. Seven specialties featured in the included work: minimally invasive and laparoscopic surgery (n=4), open and basic surgical skills (n=2), neurosurgery (n=2), neurovascular and cerebrovascular surgery (n=1), otology (n=1), and spine surgery (n=1). Full characteristics of each included study are provided in Table 2.

**Table 2.** Characteristics of included studies (n=11), covering 7 surgical specialties, published between 2021 and 2025, encompassing 347 participants with novice or limited prior procedural experience.

Author (year)	Specialty/procedure	n	Participants	Design	AR^a^ platform	Comparator	Primary outcome
Wolf et al (2021) [1]	ECMO^b^ cannulation	21	Medical students (Y3-Y4)	Crossover RCT^c^	HoloLens 2 step-by-step AR guide	Conventional SOP^d^ (paper/video)	Error count; UEQ^e^
Liu et al (2024) [2]	Neurosurgery: MCA^f^ aneurysm localization	8	Neurosurgery residents (PGY1-4; <5 aneurysm cases)	Prospective cohort	Magic Leap One + Brainlab HUD^g^ (Zeiss Kinevo 900)	2D imaging review only	Aneurysm localization deviation (mm)
Cizmic et al (2023) [3]	Laparoscopic cholecystectomy (ex vivo × 10)	40	Medical students (Y3-Y6)	Parallel RCT	iSurgeon AR telestration	Verbal guidance only	Cumulative GOALS^h^/OSATS^i^; complications; CVS^j^ achievement
Felinska et al (2023) [4]	Laparoscopic basic tasks + ex vivo cholecystectomy	40	MIS^k^-naive medical students	Crossover RCT	iSurgeon + Pupil Core eye-tracking	Verbal guidance only	Gaze latency, errors, OSATS, NASA-TLX^l^
Lopes et al (2022) [5]	Basic open surgical skills: suturing (x 5 types)	20	Medical students (no prior suturing experience)	Parallel RCT (evaluator-blinded)	Vuzix M300XL smart glasses + remote telestration	Traditional on-site teaching	Independent performance time; mOSATS score
Van Gestel et al (2021) [6]	Neurosurgery: EVD^m^ placement (phantom)	16	Medical students (no prior EVD experience)	Parallel RCT (pre/post training)	HoloLens 1 + IR^n^ inside-out tracking	Freehand technique	Mean target error (mm); mKS^o^ grade
Wild et al (2022) [7]	Laparoscopic basic skills + ex vivo cholecystectomy	60	Laparoscopic novices (medical students Y3-Y6)	Crossover RCT	iSurgeon AR telestration	Verbal guidance only	Total training time; GOALS/OSATS; complications; NASA-TLX
Hadida Barzilai et al (2025) [8]	Otology: mastoidectomy drilling (3D-printed model)	21	Medical students (clinical clerkship; no prior temporal bone drilling)	Parallel RCT	HoloLens 2 + D2P QR-code registration	Anatomy review + instructional video + dissection manual	Modified Welling Scale (mWS, /25)
Nagayo et al (2022) [9]	Open surgery: subcuticular interrupted suturing	38	Medical students (suturing novices)	Parallel RCT (evaluator-blinded)	HoloLens 2 self-training (3D expert procedure replication)	2D instructional video	Global rating; task-specific suturing scores
Kong et al (2025) [10]	Spine surgery: pedicle screw placement (L2 lumbar model)	4 surgeons / 80 screws	1 experienced surgeon + 1 novice per group	Prospective cohort	HoloLens 2 + Vuforia 3D registration	Freehand technique	Linear deviation (mm); angular deviation; Gertzbein-Robbins accuracy
Dodier et al (2024) [11]	Neurosurgery: intracranial aneurysm clipping (perfused phantom)	9 residents	Neurosurgery residents (PGY1-6; no prior clipping as lead surgeon)	3-arm RCT	HoloLens 1 holographic AR clipping simulation (SOFA^p^)	No interim training or video review only	Occlusion rate (Raymond-Roy class 1); clipping attempts; wrist tremor

a AR: augmented reality.

b ECMO: extracorporeal membrane oxygenation.

c RCT: randomized controlled trial.

d SOP: standard operating procedure.

e UEQ: User Experience Questionnaire.

f MCA: middle cerebral artery.

g HUD: heads-up display.

h GOALS: Global Operative Assessment of Laparoscopic Skills.

i OSATS: Objective Structured Assessment of Technical Skills.

j CVS: critical view of safety.

k MIS: minimally invasive surgery.

l NASA-TLX: NASA Task Load Index.

m EVD: external ventricular drain.

n IR: infrared.

o mKS: Modified Kakarla Scale.

p SOFA: Simulation Open Framework Architecture.

### AR Technology Platforms

The Microsoft HoloLens (versions 1 and 2) was the most commonly used AR platform, applied in 5 of the 11 included studies [1,6,8,9,11]. The HoloLens is an optical see-through HMD capable of projecting holographic content into the user’s visual field while preserving contact with the physical environment. Three studies used the iSurgeon telestration system, a laparoscopic screen-based AR device that projects a real-time feed of the instructor’s hand gestures onto the operative monitor [3,4,7]. The Magic Leap One combined with the Brainlab Mixed Reality Viewer and intraoperative heads-up display was used in one neurosurgical study [2]. The Vuzix M300XL smart glasses were evaluated in one suturing study [5]. Kong et al [10] used the HoloLens 2 in combination with custom surgical guides and the Vuforia 3D registration software for spinal navigation, and Dodier et al [11] used the HoloLens 1 to deliver holographic finite-element simulation of aneurysm clipping.

### Risk of Bias

Risk of bias assessment findings are summarized narratively below. Among the 9 RCTs, 6 were assessed as having some concerns regarding randomization or blinding processes [1,3,4,7,9,11]; full blinding of participants and instructors to group allocation is inherently unfeasible in AR training studies, representing a structural limitation of all trials in this field. The remaining 3 RCTs [5,6,8] were assessed as low risk across all domains. The 2 nonrandomized studies [2,10] were assessed using ROBINS-I and rated as moderate risk, reflecting their small sample sizes and lack of formal randomization, though both did use internal controls. Outcome assessment blinding was reported in 4 studies [5,6,8,9], which goes some way toward reducing detection bias. None of the included studies reported any long-term follow-up or skill retention data—a gap that runs consistently across the entire evidence base.

### Narrative Synthesis of Outcomes

#### Domain 1: Technical Accuracy and Procedural Performance

Of the 11 included studies, 6 provided direct evidence of AR improving objective technical accuracy. The most pronounced effects were observed in procedural tasks with a strong visuospatial component. Van Gestel et al [6] demonstrated that untrained medical students using AR guidance for external ventricular drain (EVD) placement on a phantom model achieved a mean target error of 11.9 mm, compared with 19.9 mm for the untrained freehand group (*P*=.003). Critically, untrained AR-guided performance matched that of trained freehand performers, indicating that AR effectively compressed the procedural learning curve. The quality of EVD placement was also significantly superior in the AR group (59.4% vs 25% Modified Kakarla Scale grade 1, *P*=.005) [6].

Hadida Barzilai et al [8] reported significantly superior overall mastoidectomy performance in the AR group (median Modified Welling Scale 19.5/25) compared with controls (12/25; *P*=.001), with significant advantages on 6 of 8 subscales including mastoidectomy margin definition, sinodural angle, and tegmen exposure. The AR group’s score exceeded published novice benchmark values.

Liu et al [2] demonstrated that AR-assisted visuospatial training significantly reduced aneurysm localization deviation among neurosurgical residents, from 8.1 mm at AR Test 1 to 2.7 mm at AR Test 2 (*P*<.001). Crucially, this improvement was retained in the final test conducted without any AR assistance (AR group: 2.1 mm vs control: 5.9 mm; *P*<.001), confirming durable skill acquisition rather than performance scaffolding alone.

Kong et al [10] demonstrated AR’s equalizing effect on novice-expert performance disparity in pedicle screw placement: overall AR accuracy 95% versus 77.5% freehand (*P*<.05 for both linear and angular deviation). In contrast, Nagayo et al [9] found no significant difference between AR and video-based self-training in suturing skill improvement (global rating: *P*=.54; task specific: *P*=.91), and Lopes et al [5] similarly found no significant difference in mOSATS scores, although the telestration group performed tasks significantly faster when working independently (1393 s vs 1679 s; *P*=.04). These findings represent noninferiority rather than inferiority of AR.

#### Domain 2: Error Reduction and Procedural Safety

AR was consistently associated with reduced procedural errors across all 5 studies that measured this outcome. Felinska et al [4] demonstrated the most dramatic reduction, with AR-instructed trainees making a mean of 0.18 errors per task compared with 1.94 for the verbal instruction group (*P*<.01; ηp²=0.92), representing a tenfold reduction in error rate. Complementary eye-tracking data revealed the mechanism: AR reduced gaze latency from 2.04 to 0.21 seconds (*P*<.01; ηp²=0.95), confirming that AR telestration functions by directing trainees’ visual attention to operationally relevant structures more rapidly and precisely than verbal instruction [4].

Wolf et al [1] found that AR-based extracorporeal membrane oxygenation cannulation instructions resulted in a 66% reduction in knowledge-related errors for the more complex second procedure (18 vs 53 errors; *P*<.05), while handling errors were unchanged, suggesting that AR’s error-reducing effect is specifically mediated by improved information accessibility and cognitive offloading. Cizmic et al [3] reported that the iSurgeon group incurred significantly fewer total complications and achieved the critical view of safety in 79.5% of procedures compared with only 41.4% in the verbal guidance group (*P*≤.001). Wild et al [7] similarly reported a significant reduction in complication rates with AR telestration (13.3% vs 40%; *P*=.02).

#### Domain 3: Learning Trajectory and Skill Acquisition

Four studies provided explicit evidence regarding AR’s effect on the learning trajectory. Liu et al [2] showed a steep decline in localization deviation across successive AR test blocks while the control groups remained relatively flat, indicating an accelerated learning curve rather than a simple one-time performance advantage. Cizmic et al [3] provided longitudinal evidence across 10 cholecystectomy sessions, demonstrating that the AR telestration group maintained consistently higher GOALS (Global Operative Assessment of Laparoscopic Skills) and OSATS (Objective Structured Assessment of Technical Skills) scores from the first session onwards, with the performance gap not narrowing over time. Van Gestel et al [6] finding that untrained AR performers matched trained freehand performers is perhaps the most striking demonstration of learning curve compression in this review. Dodier et al [11] found that only the video-plus-AR cohort achieved a statistically significant improvement in aneurysm occlusion rate between the first and final sessions (67% to 93%; *P*=.05), demonstrating that AR adds value beyond video review alone for complex microsurgical skill acquisition.

#### Domain 4: Cognitive Load and Gaze Efficiency

Three studies measured cognitive load using validated instruments. Felinska et al [4] reported significantly lower NASA Task Load Index scores during basic laparoscopic tasks with AR telestration compared with verbal instruction (mean 50 [SD 21] vs mean 56 [SD 22]; *P*<.01), alongside a lower objective blink rate. Wild et al [7] reported that participants found AR training significantly less mentally demanding (mean 33.3 [SD 14.8] vs mean 48.9 [SD 14.3]; *P*<.001) and less physically demanding (mean 35.1 [SD 13.8] vs mean 38.1 [SD 13.3]; *P*=.002). Liu et al [2] noted that the AR group took significantly longer to complete tasks in early test phases (*P*=.003), attributed to the additional cognitive processing required to colocate spatial AR hologram information with physical understanding, an effect that decreased as participants became more familiar with the AR system.

#### Domain 5: Operational Efficiency and Procedure Time

The effect of AR on procedure and training time was mixed across studies. Wild et al [7] reported the most pronounced efficiency gain: total laparoscopic training time was reduced by 29.8% with AR telestration (mean 1163 [SD 275] vs mean 1658 [SD 375] seconds; *P*<.001). Lopes et al [5] reported significantly faster independent suture completion in the AR group (1393 vs 1679 seconds; *P*=.04). For more complex procedures, no significant differences in total operative time were observed (mean 79.6 [SD 25.7] vs mean 84.5 [SD 33.2] minutes; *P*=.09), suggesting that AR’s efficiency benefits are most readily detectable in discrete, structured tasks (Table 3).

**Table 3.** Summary of primary outcomes and key quantitative findings across included studies (n=11), covering participants enrolled in surgical and procedural training programs across 7 specialties, 2021‐2025.

Author (year)	Primary outcome	AR^a^ result	Control result	*P* value	Key interpretation
Wolf et al (2021) [1]	Error count (procedure 2)	18 knowledge errors	53 knowledge errors	<.05	66% reduction in knowledge errors; handling errors unchanged—AR reduces cognitive errors specifically
Liu et al (2024) [2]	Localization deviation (mm)	Mean 2.7 (SD 1.0) mm (AR test 2); 2.1 mm (final test without AR)	Mean 5.8 (SD 4.1) mm; 5.9 mm (final test)	.01; <.001	AR accelerates visuospatial learning curve; improvement retained in final unassisted test
Cizmic et al (2023) [3]	GOALS^b^; OSATS^c^; CVS^d^	GOALS 17.3; OSATS 50.8; CVS 79.5%	GOALS 16.0; OSATS 41.2; CVS 41.4%	<.001 (all)	AR telestration maintains higher performance trajectory across 10 LCs^e^; CVS achievement nearly doubled
Felinska et al (2023) [4]	Error count; gaze latency; OSATS	0.18 errors; 0.21 s gaze latency	1.94 errors; 2.04 s gaze latency	<.01 (all)	Tenfold error reduction; gaze guidance mechanism confirmed by eye-tracking; reduced NASA-TLX^f^
Lopes et al (2022) [5]	Independent task time; mOSATS	1393 s total; mOSATS trend higher	1679 s total	.04 (time)	AR telestration produces faster independent performance; quality comparable—viable alternative to on-site teaching
Van Gestel et al (2021) [6]	Mean target error (mm); mKS^g^ grade 1	11.9 mm; 59.4% grade 1	19.9 mm; 25% grade 1	.003; .005	AR eliminates procedural learning curve for EVD^h^ placement; untrained AR matches trained freehand
Wild et al (2022) [7]	Training time; GOALS/OSATS; complications	1163 s; GOALS 21; OSATS 67; 13.3% complications	1658 s; GOALS 18; OSATS 61; 40% complications	<.001; .007; .015; .020	29.8% training time; significant quality and safety improvement; reduced NASA-TLX
Hadida Barzilai et al (2025) [8]	Modified Welling Scale (/25)	19.5/25 (median)	12/25 (median)	.001	7.5-point advantage; AR group exceeds published novice benchmarks; 6/8 subscales significant
Nagayo et al (2022) [9]	Global rating (GR); task-specific (TS) scores	GR 16.03; TS 15.03 (posttest)	GR 15.5; TS 15.11 (posttest)	.54; .91 (NS^i^)	Noninferior to video self-training; AR rated more useful for 3D instrument motion (*P*=.02)
Kong et al (2025) [10]	Linear deviation; angular deviation; Gertzbein-Robbins accuracy	Novice: 1.73 mm / 2.87 degrees; 90% accuracy	Novice: 5.25 mm / 7.15 degrees; 70% accuracy	<.05 (all)	AR equalizes novice-expert performance gap; overall accuracy 95% vs 77.5% freehand
Dodier et al (2024) [11]	Aneurysm occlusion rate (Raymond-Roy class 1)	67%-93% (video + AR cohort)	67%-73% (video only); stable (control)	.046	Only video + AR cohort achieved significant occlusion improvement; AR adds value beyond video alone

a AR: augmented reality.

b GOALS: Global Operative Assessment of Laparoscopic Skills.

c OSATS: Objective Structured Assessment of Technical Skills.

d CVS: critical view of safety.

e LC: laparoscopic cholecystectomy.

f NASA-TLX: NASA Task Load Index.

g mKS: Modified Kakarla Scale.

h EVD: external ventricular drain.

i NS: not significant.

## Discussion

### Principal Findings

This systematic review synthesizes evidence from 11 contemporary studies (2021‐2025) evaluating AR’s impact on the objective technical performance of surgical trainees. The principal finding is that AR demonstrates a consistent, measurable positive effect on technical performance, most strongly in domains requiring visuospatial reasoning, spatial anatomical understanding, and procedural accuracy. Of the 11 studies, 9 reported at least one significant improvement in an objective technical performance metric. The 2 studies that did not demonstrate AR superiority [5,9] nonetheless showed noninferiority, with AR-trained groups performing equivalently to comparators on skill quality metrics while achieving time advantages in independent performance. The absence of superiority in lower-stakes basic skills tasks is not evidence of inefficacy; it may reflect a ceiling effect in tasks where traditional instruction is already adequate for novice performance.

Across the body of evidence, the most robust performance advantages were observed in tasks with a strong visuospatial or spatial navigation component: EVD placement [6], aneurysm localization [2], mastoidectomy drilling [8], and pedicle screw placement [10]. This pattern is theoretically coherent: AR’s capacity to render 3D anatomical structures in the trainee’s visual field directly addresses a fundamental cognitive challenge in procedural surgery: the mental reconstruction of volumetric anatomy from 2D imaging data.

### Interpretation and Comparison With Prior Literature

The findings of this review are consistent with, and substantially extend, the conclusions of prior systematic reviews. Abu Halimah et al [15] and Xiong et al [16] identified broad potential for AR in surgical skills training, but their reviews included older studies with heterogeneous definitions of AR and outcomes. By restricting our scope to post-2020 studies with objective outcomes and clear AR definitions, we provide a more precise assessment applicable to current training environments. Importantly, this review positions AR and VR as complementary rather than competitive modalities, a distinction emphasized in the literature [11,24].

The observed expertise reversal pattern, AR providing the greatest benefit to novices with diminishing returns at higher levels of proficiency, is consistent with predictions from both cognitive load theory [12] and the expertise reversal effect [14]. In Kong et al [10], AR navigation essentially equalized the novice-expert performance gap. This pattern has direct implications for curriculum design: AR-assisted training may be most efficiently used during the early stages of procedural learning, with progressive withdrawal of AR guidance as competency develops, a strategy consistent with the scaffolding framework in educational theory [25,26].

The gaze-guidance mechanism elucidated by Felinska et al [4] provides the most direct experimental evidence for the cognitive mechanism underpinning AR’s training benefit. By demonstrating that AR telestration reduced gaze latency tenfold and aligned trainee gaze with expert gaze, this study demonstrates that AR’s error-reducing effect is mediated by directing visual attention to operationally relevant anatomical regions more efficiently than verbal instruction.

Dodier et al [11] finding that only the combined video-plus-AR cohort achieved a significant improvement in aneurysm occlusion rate (67% to 93%; *P*=.05) is particularly noteworthy. The holographic AR clipping simulation allowed residents to test different clipping strategies on the exact same patient-specific anatomy as the physical phantom, a form of deliberate practice that is impossible with traditional simulation.

### Limitations

Several limitations of the constituent studies and of this review must be acknowledged. The most fundamental limitation is heterogeneity: the 11 included studies span 7 surgical specialties, use 6 distinct AR platforms, and measure outcomes using a wide variety of instruments, precluding statistical synthesis. Sample sizes were consistently small (range: 8‐60 participants), limiting statistical power. Publication bias cannot be excluded.

None of the 11 included studies reported long-term follow-up of skill retention or assessed the transfer of AR-trained skills to the real operating room or clinical environment. This is perhaps the most significant gap in the current evidence base. Device-related limitations were noted across several studies, including physical discomfort associated with prolonged HMD use [6] and interface familiarization time across HoloLens-based studies. The risk of bias assessment found some concerns in 6 of 11 RCTs, predominantly related to allocation concealment and blinding, which is an inherent structural limitation of AR training trials rather than a correctable methodological weakness. The review was not prospectively registered, which is acknowledged as a limitation.

One study for which full text could not be obtained despite institutional access, interlibrary loan request, and direct author contact was excluded; this represents 1 of 29 full-text articles reviewed (3.4%) and, given the consistency of findings across the 11 included studies, is unlikely to materially alter the direction of the conclusions.

### Future Research Directions

The current evidence points toward several concrete priorities for future work. Most pressing is the need for adequately powered multicenter randomized trials across the more promising AR platforms and specialty domains, using standardized outcome measures that would actually allow findings to be compared across studies—something the current literature makes difficult. Alongside this, longitudinal studies with skill-retention assessments at 3, 6, and 12 months posttraining are needed to establish whether the performance gains associated with AR hold over time or fade once the technology is removed. Transfer studies examining whether AR-trained skills translate meaningfully into clinical performance are a logical next step that the field has yet to take seriously. Finally, cost-effectiveness analyses will matter enormously for any health system considering curriculum-level adoption—the training benefit needs to be weighed against the real costs of hardware, software, and implementation, and that work has not yet been done.

### Conclusions

This review found consistent evidence that AR improves technical performance in surgical novices—reduced procedural errors, better accuracy, faster progression along the learning curve, and lower cognitive load, particularly in tasks with high visuospatial demands. The expertise reversal pattern that emerged across multiple studies is worth taking seriously: AR appears to deliver its greatest benefit during the early, high-error phase of skill acquisition, with returns diminishing as experience accumulates. That finding has practical implications for how AR should be used—not as a permanent scaffold, but as a targeted intervention in early training, with guidance progressively withdrawn as competency develops.

What this review cannot claim is that the evidence is mature. Sample sizes are small, platforms vary enormously, outcome measures are inconsistent, and no study has yet examined whether skills are retained or transferred to real clinical settings. AR shows genuine promise as an adjunct within structured surgical curricula—not a replacement for expert mentorship or traditional teaching, but something that adds real value when used thoughtfully alongside them. Turning that promise into confident implementation guidance will require the kind of rigorous, large-scale, longitudinal work that the field has not yet produced.

## Supplementary material

10.2196/71572 Multimedia Appendix 1 Full electronic search strategies.

10.2196/71572 Checklist 1 PRISMA 2020 checklist.

## Abbreviations

AR: augmented reality
EVD: external ventricular drain
GOALS: Global Operative Assessment of Laparoscopic Skills
HMD: head-mounted display
MeSH: Medical Subject Headings
MR: mixed reality
NASA-TLX: NASA Task Load Index
OSATS: Objective Structured Assessment of Technical Skills
PICO: population, intervention, comparison, outcome
PRISMA: Preferred Reporting Items for Systematic Reviews and Meta-Analyses
RCT: randomized controlled trial
RoB 2: Cochrane Risk of Bias 2 Tool
ROBINS-I: Risk of Bias in Nonrandomized Studies of Interventions
SWiM: Synthesis Without Meta-Analysis
VR: virtual reality
