# Correction: Augmented Reality in Surgical Training: Systematic Review of Its Impact on Technical Performance in Surgical Trainees

**DOI:** 10.2196/106576

**Published:** 2026-07-23

**Authors:** Mahmoud El Ashry, Ahmed El Ashry, Hamza Khalique, Yahye Abdalle, Thomas Yeung

**Affiliations:** 1 Bristol Medical School, University of Bristol, Beacon House, Queens Road, Bristol, BS8 1QU, United Kingdom, 44 7448827361; 2 ENT/Otolaryngology Department, University Hospitals of North Midlands NHS Trust, Stoke-on-Trent, United Kingdom

In “Augmented Reality in Surgical Training: Systematic Review of Its Impact on Technical Performance in Surgical Trainees” [1], the authors made one correction.

The fourth author’s name was amended to read from the following:

*Yahyea Abdalle*

The author’s name now reads:

Yahye Abdalle

The correction will appear in the online version of the paper on the JMIR Publications website, together with the publication of this correction notice. Because this was made after submission to PubMed, PubMed Central, and other full-text repositories, the corrected article has also been resubmitted to those repositories.
