# Supplementary material for: Augmented Reality in Surgical Training: Systematic Review of Its Impact on Technical Performance in Surgical Trainees
Source: JMIR Med Educ. 2026 Jun 3;12:e71572. doi: 10.2196/71572 (PMC13233012; doi:10.2196/71572)
Supplement: Multimedia Appendix 1 [file mededu-v12-e71572-s001.docx]

# Multimedia Appendix 1: Full Electronic Search Strategies (PRISMA-S)

The following search strategies were executed on April 24, 2026, with results filtered to the eligibility window of January 1, 2020 to September 15, 2025, across all six databases. Strategies are presented in their database-specific syntax in accordance with the PRISMA-S reporting extension [20].

## 1. PubMed (MEDLINE via NLM)

*("Augmented Reality"[MeSH] OR "augmented reality"[tiab] OR "mixed reality"[tiab] OR "head-mounted display"[tiab] OR "HMD"[tiab] OR "heads-up display"[tiab] OR "HUD"[tiab] OR "holographic"[tiab] OR "telestration"[tiab] OR "HoloLens"[tiab] OR "Magic Leap"[tiab] OR "smart glasses"[tiab] OR "optical see-through"[tiab] OR "AR-assisted"[tiab] OR "AR-guided"[tiab] OR "AR-enhanced"[tiab]) AND ("Education, Medical"[MeSH] OR "Education, Medical, Graduate"[MeSH] OR "Clinical Competence"[MeSH] OR "Simulation Training"[MeSH] OR "Internship and Residency"[MeSH] OR "surgical train*"[tiab] OR "surgical educat*"[tiab] OR "surgical skill*"[tiab] OR "surgical simulat*"[tiab] OR "procedural train*"[tiab] OR "procedural skill*"[tiab] OR "resident*"[tiab] OR "novice*"[tiab] OR "trainee*"[tiab] OR "medical student*"[tiab] OR "laparoscopic train*"[tiab] OR "minimally invasive train*"[tiab] OR "neurosurgery train*"[tiab]) AND ("learning curve"[tiab] OR "technical performance"[tiab] OR "skill acquisition"[tiab] OR "psychomotor"[tiab] OR "accuracy"[tiab] OR "proficiency"[tiab] OR "competency"[tiab] OR "OSATS"[tiab] OR "GOALS"[tiab] OR "error rate"[tiab] OR "performance score"[tiab] OR "procedure time"[tiab]) AND ("2020/01/01"[PDat]:"2025/09/15"[PDat])*

## 2. Ovid MEDLINE

*1. exp Augmented Reality/ OR (augmented reality OR mixed reality OR head-mounted display OR heads-up display OR telestration OR HoloLens OR Magic Leap OR smart glass* OR holographic).ti,ab. 2. exp Education, Medical/ OR exp Clinical Competence/ OR exp Simulation Training/ OR exp Internship and Residency/ OR (surgical train* OR surgical skill* OR surgical educat* OR procedural train* OR resident* OR novice* OR trainee* OR medical student*).ti,ab. 3. (learning curve OR technical performance OR skill acquisition OR psychomotor OR accuracy OR proficiency OR competency OR OSATS OR GOALS OR error rate OR performance score).ti,ab. 4. 1 AND 2 AND 3. 5. Limit 4 to (yr="2020-2025" AND English language)*

## 3. Embase (Ovid)

*1. exp augmented reality/ OR (augmented reality OR mixed reality OR head-mounted display OR HoloLens OR telestration OR smart glass* OR holographic OR heads-up display).ti,ab. 2. exp medical education/ OR exp clinical competence/ OR exp simulation/ OR (surgical train* OR surgical skill* OR procedural train* OR resident* OR novice* OR trainee* OR medical student*).ti,ab. 3. (learning curve OR technical performance OR skill acquisition OR accuracy OR proficiency OR OSATS OR GOALS OR error rate).ti,ab. 4. 1 AND 2 AND 3. 5. Limit 4 to (yr 2020-2025 AND English language AND article)*

## 4. IEEE Xplore

*("augmented reality" OR "mixed reality" OR "head-mounted display" OR "HoloLens" OR "Magic Leap" OR "telestration" OR "smart glasses" OR "holographic display") AND ("surgical training" OR "surgical education" OR "surgical skills" OR "medical training" OR "procedural training" OR "laparoscopic training" OR "simulation" OR "trainee" OR "resident" OR "novice" OR "medical student") AND ("performance" OR "accuracy" OR "learning curve" OR "skill acquisition" OR "error" OR "OSATS" OR "GOALS") [2020-2025]*

## 5. Scopus

*TITLE-ABS-KEY ( ("augmented reality" OR "mixed reality" OR "head-mounted display" OR "HoloLens" OR "telestration" OR "smart glasses" OR "holographic" OR "heads-up display") AND ("surgical training" OR "surgical education" OR "surgical skill*" OR "procedural training" OR "laparoscopic training" OR "simulation training" OR "resident*" OR "trainee*" OR "novice*" OR "medical student*") AND ("technical performance" OR "learning curve" OR "skill acquisition" OR "accuracy" OR "error" OR "OSATS" OR "GOALS" OR "proficiency") ) AND PUBYEAR > 2019 AND PUBYEAR < 2026 AND ( LIMIT-TO ( DOCTYPE,"ar" ) ) AND ( LIMIT-TO ( LANGUAGE,"English" ) )*

## 6. Web of Science (Core Collection)

*TS=("augmented reality" OR "mixed reality" OR "HoloLens" OR "head-mounted display" OR "telestration" OR "smart glasses" OR "holographic") AND TS=("surgical training" OR "surgical education" OR "surgical skill*" OR "laparoscopic training" OR "procedural training" OR "resident*" OR "trainee*" OR "novice*" OR "medical student*") AND TS=("technical performance" OR "learning curve" OR "skill acquisition" OR "accuracy" OR "OSATS" OR "GOALS" OR "error" OR "proficiency") AND PY=(2020-2025) AND LA=(English)*
