# Supplementary material for: Augmented Reality in Surgical Training: Systematic Review of Its Impact on Technical Performance in Surgical Trainees
Source: JMIR Med Educ. 2026 Jun 3;12:e71572. doi: 10.2196/71572 (PMC13233012; doi:10.2196/71572)
Supplement: Checklist 1 [file mededu-v12-e71572-s002.docx]

# PRISMA 2020 Expanded Checklist

The following PRISMA 2020 checklist documents compliance with each reporting item. Section references correspond to the main manuscript.

| **#** | **PRISMA 2020 Item** | **Location in Manuscript** | **Reported** |
| --- | --- | --- | --- |
| 1 | Identify the report as a systematic review | Title; Abstract | Yes |
| 2 | Provide a structured summary including background, objectives, methods, results, conclusions | Abstract | Yes |
| 3 | Describe the rationale for the review in the context of what is already known | Background (Introduction) | Yes |
| 4 | Provide an explicit statement of objectives or questions addressed by the review | Background (final paragraph); Methods (Study Design) | Yes |
| 5 | Specify whether a review protocol was registered or not, where it can be accessed, and any amendments | Methods: Study Design and Registration | Yes (not registered; a priori protocol followed internally) |
| 6 | Specify all eligibility criteria and rationale for chosen criteria | Methods: Eligibility Criteria; Table 1 | Yes |
| 7 | Describe all information sources used, including any automation tools used, and the date each was last searched | Methods: Information Sources | Yes |
| 8 | Present the full search strategies for all databases, including any filters applied | Methods: Search Strategy; Multimedia Appendix 1 (PRISMA-S compliant) | Yes |
| 9 | Specify methods used to select studies - screening process, number of reviewers, resolution of discrepancies | Methods: Study Selection | Yes |
| 10 | Specify methods for data extraction, including any processes for obtaining or confirming data | Methods: Data Collection Process and Items | Yes |
| 11 | List and define all outcomes for which data were sought | Methods: Synthesis Methods; Results: Domains 1–5 | Yes |
| 12 | Describe methods of assessing risk of bias | Methods: Risk of Bias Assessment | Yes |
| 13 | Describe the methods of synthesis used | Methods: Synthesis Methods (SWiM) | Yes |
| 14 | Describe the results of the search and selection process | Results: Study Selection; Figure 1 (PRISMA flow diagram) | Yes |
| 15 | Cite each potentially eligible study and explain why it was excluded | Results: Study Selection; reasons documented per stage | Yes |
| 16 | Cite each included study and present its characteristics | Results: Study Characteristics; Table 2 | Yes |
| 17 | Present assessments of risk of bias for each included study | Results: Risk of Bias | Yes |
| 18 | For all outcomes, present, for each study, the extracted results | Results: Table 3; narrative synthesis Domains 1–5 | Yes |
| 19 | Provide a synthesis of results from the studies included in the synthesis | Results: Domains 1–5 | Yes |
| 20 | Report any assessments of certainty (or confidence) in the body of evidence | Discussion: Limitations | Yes |
| 21 | Provide a general interpretation of the results | Discussion: Principal Findings; Interpretation | Yes |
| 22 | Provide information about any potential sources of bias in the review process | Discussion: Limitations | Yes |
| 23 | Describe the evidence relating to each major finding of the review | Discussion: Domains 1–5 mapped to Principal Findings | Yes |
| 24 | Provide an overall interpretation of the findings | Discussion: Conclusions | Yes |
| 25 | Describe any funding sources for the review, and the role of the funders | Funding / COI / Declarations section | Yes |
| 26 | Report on any potential conflicts of interest | Conflicts of Interest statement | Yes |
| 27 | Report whether the review protocol was prospectively registered | Methods: Study Design and Registration | Yes |
